# Clinical Evaluation of Adrenal Incidentaloma: The Experience of a Referral Center

**DOI:** 10.3390/biomedicines12081910

**Published:** 2024-08-20

**Authors:** Luigi Petramala, Francesco Circosta, Luca Marino, Edoardo Palombi, Maria Ludovica Costanzo, Adriana Servello, Gioacchino Galardo, Claudio Letizia

**Affiliations:** 1 Department of Translational and Precision Medicine, “Sapienza” University of Rome, 00185 Rome, Italy; luigi.petramala@uniroma1.it; 2 Department of Clinical, Internal, Anesthesiological and Cardiovascular Sciences, “Sapienza” University of Rome, 00185 Rome, Italy; francesco.circosta@uniroma1.it (F.C.); marialudovica.costanzo@uniroma1.it (M.L.C.); claudio.letizia@uniroma1.it (C.L.); 3 Emergency Medicine Unit, Department of Emergency-Acceptance, Critical Areas and Trauma, Policlinico “Umberto I”, 00185 Rome, Italy; edoardo.palombi@uniroma1.it (E.P.); adriana.servello@uniroma1.it (A.S.); g.galardo@policlinicoumberto1.it (G.G.); 4 Department of Mechanical and Aerospace Engineering, “Sapienza” University of Rome, 00185 Rome, Italy

**Keywords:** adrenal incidentaloma, dexamethasone test, hypertension, metabolic disorders

## Abstract

The number of adrenal incidentaloma (AI) cases has increased in the last few years due to the widespread use of imaging diagnostics. Management requires evaluation of the malignant nature and hormonal activity. The aim of the present study is to assess possible clinical abnormalities in 132 AI patients both at baseline and during follow-up (mean 48.6 ± 12.5 months). In all patients, demographic, anthropometric data, biochemical, metabolic and hormonal data, and 24-h ambulatory blood pressure monitoring were assessed. Mild autonomous cortisol secretions (MACS) were diagnosed in patients without signs and symptoms of overt Cushing’s syndrome and post dexamethasone (DXM) plasma cortisol concentration > 50 nmol/L (>1.8 μg/dL). Patients with overnight DXM-1 mg test positive showed higher values of diastolic blood pressure, glycemia and uric acid levels compared to patients with negative DXM test at baseline. During follow-up, the potential development of MACS in patients with nonfunctional AI showed a prevalence of 29%, though the cardiovascular and metabolic alterations were less pronounced compared to those diagnosed with MACS at baseline. Therefore, follow-ups with AI patients are useful for observing changes in clinical features.

## 1. Introduction

The increased use of imaging techniques has led to a rise in the detection of lesions affecting the adrenal glands, commonly defined as “adrenal incidentaloma” (AI), with a prevalence ranging from 3 to 10% in the adult population [1,2]. Upon discovery of an AI, it is crucial to assess both the potential malignancy of the lesion (either as a primary lesion or as a metastatic site) and any hormonal hyperactivity. Among the latter, the most common condition is a cortisol-secreting adrenal adenoma, estimated at 29% of overall AI [2]. Autonomous cortisol secretion is strongly associated with the development of systemic alterations such as arterial hypertension, diabetes mellitus, dyslipidemia, visceral obesity, sarcopenia, osteoporosis, infections, cardiovascular diseases, and cardiomyopathy [3,4,5,6,7,8,9].

Over the years, the use of the DXM test (overnight Dexamethasone 1 mg test) has become the cornerstone of the evaluation of the possible autonomous secretion of cortisol, no longer distinguishing between possible and confirmed autonomous secretion but identifying the entity called “mild autonomous cortisol secretions” (MACS) in case of plasma cortisol > 50 nmol/L (> 1.8 mg/dL) after DXM test and suppressed ACTH levels. This approach eliminates the need for further hormonal alterations of the hypothalamic-pituitary-adrenal axis (i.e., 24-h urinary cortisol excretion, circadian cortisol dosage) [2,10].

International Guidelines suggest the following scenarios: patients with cortisol < 50 nmol/L post-DXM test do not need any further evaluation or follow-up, whereas in MACS patients (cortisol > 50 nmol/L post-DXM test without a clinical picture of overt Cushing’s syndrome) the assessment of the presence of cortisol-related comorbidities is crucial to further management, deciding removal of cortisol-secreting adenoma or pharmacological treatment of risk factors; MACS patients without detectable comorbidities requiring follow-up for the development of comorbidities or clinical feature of hypercortisolism [10]. Regarding the need to carry out checks over time to evaluate the cortisol-secreting autonomy of AIs, International Guidelines recommend against repeated hormonal work-up in patients with hormonal tests within the reference range at initial evaluation unless new clinical signs of endocrine activity appear or worsening of comorbidities (hypertension, type 2 diabetes). However, some authors suggest that the DXM test, representing an inexpensive, invasive and easy tool, could be performed for a period of at least 5 years after the diagnosis of non-functional AI (NFAI) [11]. In fact, different studies have highlighted evidence of hormonal activity in patients initially identified as not hormonally active, with percentages ranging from 2–30%.

The aim of the present study was to evaluate AI patient’s the cortisol-secretion, both baseline and annually, over a five-year follow-up, focusing on the metabolic disorders and the blood pressure profile, assessed both clinically and through 24-h blood pressure monitoring.

## 2. Materials and Methods

In this retrospective study, after initial AI diagnosis, 132 patients (56 men and 76 women; the mean age of the participants was 61.7 ± 10.8 years) were enrolled and followed at the Departmental Unit of Arterial Hypertension of Policlinico Umberto I Hospital of Rome between 1 January 2016 and 31 December 2023.

Demographic, anthropometric, hemodynamic data, peripheral blood, and urine samples were collected in all patients to obtain biochemical parameters, blood glucose, lipid profile, and hormonal data. Blood pressure was clinically assessed by measuring pressure in a comfortable environment 3 times every 5 min between measurements. Moreover, a 24-h ambulatory blood pressure monitoring (ABPM) was performed in all subjects using the oscillometric technique, which involves a portable, lightweight, non-invasive monitor with a self-insufflating cuff (Spacelabs Medical, 90207, Issaquah, WA, USA). The recorded parameters with ABPM include average 24-h, daytime and nighttime systolic blood pressure (SBP), diastolic blood pressure (DBP), and mean heart rate (HR). Patients were classified as “dipper” (“dipping pattern”) if their nighttime SBP and DBP decreased by more than 10% and less than 20%. Ambulatory hypertension was defined as 24-h BP more than 130/80 mmHg.

AI was diagnosed through an abdominal computed tomography (CT) or a magnetic resonance (MR) request, not for adrenal diseases. Radiological criteria adopted to diagnose an adrenal mass not malignant include pre-contrast medium infusion Hounsfield unites ≤ 10 HU, a wash-out ≥50% in 15 min after contrasting medium infusion and regular margins morphology; we also evaluated the dimensions of the adrenal adenoma by considering the greater diameter [2]. Moreover, a complete clinical and hormonal evaluation was performed to exclude the presence of pheochromocytoma, primary hyperaldosteronism, and evident clinical signs of hypercortisolism or hyperandrogenism. From the study, we have excluded patients with known or highly suspected tumours, renal diseases or cysts, a history of extended steroid intake, and patients without complete follow-up. The diagnosis of MACS was performed in those patients without signs and symptoms of overt Cushing’s syndrome and post DXM plasma cortisol concentration above 50 nmol/L (>1.8 μg/dL), confirmed by a second determination [1,2]. Patients with a baseline negative DXM test, in which autonomous cortisol secretion was excluded, were subsequently followed up annually for up to 5 years, evaluating anthropometric, biochemical, hormonal, imaging (CT or MR) and ABPM parameters. We have excluded from the study the patients who had, in addition to cortisol, a proven baseline autonomous hormone production. ACTH was measured with radioimmunoassay method (RIA), reference range 10–90 pg/mL, inter/intra-assay CV 8.3/6.2; plasma cortisol was measured with CMIA (Heterogenous chemiluminescent microparticle immunoassay), reference range 266–720 nmol/L, inter/intra-assay CV 5.5/4.5; UFC was measured with RIA, reference range 38–208 nmol/24 h, inter/intra-assay CV 5.5/4.5. All hormone assays were performed with commercially available kits. Plasma and urinary cortisol were measured through the Architect Cortisol Reagent Kit produced for Abbott by Fisher Scientific Company LLC, 8365 Valley Pike, Middletown, VA, USA.

This study was conducted in accordance with the Declaration of Helsinki guidelines and was approved by a local ethical committee. The study design was clearly written in layperson language and provided to each study participant. Written informed consent was obtained from all patients. Clinical data were obtained from routine clinical practice, so we have requested consent from the Local Ethical Committee of the Department of Clinical, Internal, Anesthesiological and Cardiovascular Sciences, “Sapienza” University of Rome, Italy.

The data are expressed as mean value and standard deviation (SD). Differences between numerical data were assessed by the Mann–Whitney U test for two-sample comparison or by one-way analysis of variance (ANOVA), which applied the Fisher least significant difference post hoc test for multiple comparisons. Chi^2 statistics were used to assess differences between categorical variables. *p* values less than 0.05 were taken as statistically significant. Statistical analysis was performed using dedicated statistical software SPSS (Statistical Package for Social Sciences, software, version 24; SPSS Inc, Chicago, IL, USA).

## 3. Results

**Figure 1:**
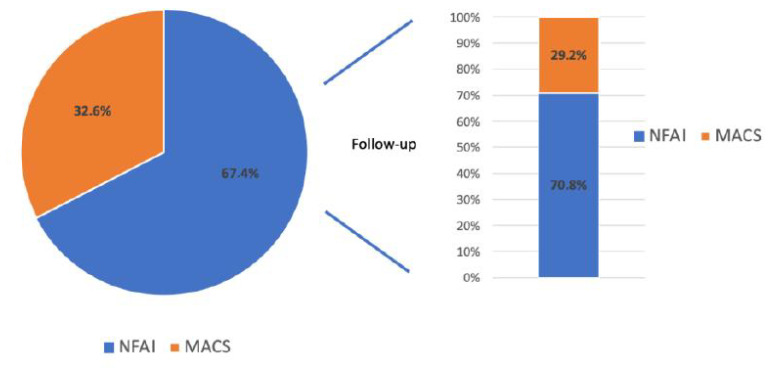
Prevalence of non-functional adrenal incidentaloma (NFAI) and mild autonomous cortisol secretions (MACS) at baseline and follow-up.

In the present study, we enrolled 132 AI patients (56 males and 76 females, mean age 61.7 ± 10.8 years) (Table 1). The patients were divided into 2 groups according to the baseline response to the DXM test: 89 patients (67.4%) were found negative (mean age 61.6 ± 11.5 years) while 43 resulted positive (32.6%) (mean age 61.8 ± 9.4 years) (Figure 1). In these two groups, no significant differences were found between anthropometric parameters such as the body mass index (BMI) and waist circumference (WC), while in subjects with positive DXM Test at baseline, we found higher mean diastolic blood pressure (DBP), clinically evaluated, respect to negative DXM Test (80.6 ± 9.9 mmHg vs 85.6 ± 8.47 mmHg; *p* < 0.05). Regarding biochemical data, at baseline, patients with a positive DXM test showed higher mean blood glucose levels (94.6 ±19.8 mg/dL vs 97.4 ± 18.7 mg/dL; <0.05) and uric acid (4.7 ± 1.6 mg/dL vs 5.4 ± 1.7 mg/dL; <0.05), respectively (Table 2). In Table 3, we have reported hormonal data, and a maximum diameter of AI evaluated by imaging techniques (TC or RMN). No significant differences in the maximum dimensions of the AI were found between patients who tested positive or negative at the DXM Test at baseline (27.1 ± 7.3 mm vs 27.5 ± 7.6 mm).

The data obtained from ABPM (Table 4) confirmed higher blood pressure load regarding the diastolic component of arterial hypertension, assessed in the 24 h (75.9 ± 9.6 mmHg vs 79.7 ± 10 mmHg; *p* < 0.05), in the daytime period (78.9 ± 10.3 mmHg vs 81.9 ± 11.2 mmHg; *p* < 0.05) and nighttime period (67.8 ± 8.5 mmHg vs 72.4 ± 10.8 mmHg; *p* < 0.05).

The patients with baseline negative DXM test were annually followed for at least 5 years, and at the end of the observation period, 29.2% of subjects resulted positive for the DXM test (at least two consecutive determinations) (Table 3); the average time of onset of cortisol secretion autonomy was 48.6 ± 12.5 months. During follow-up, no differences in the maximum dimensions of the AI were found between positive and negative patients at the DXM Test at baseline (28.3 ± 8.8 mm vs 28.7 ± 9.2 mm).

Interestingly, compared to the patients negative on the DXM Test at the end of follow-up, the positive patients showed higher diastolic blood pressure values, assessed both clinically (81.6 ± 10.5 mmHg vs 83.7 ± 9.7 mmHg; *p* < 0.05) and through the ABPM (24-h: 71.3 ± 6.9 mmHg vs 76.6 ± 8.8 mmHg; <0.05; daytime: 73.9 ± 7.3 mmHg vs 79.2 ± 8.9 mmHg, *p* < 0.05; nighttime: 64.5 ± 7.2 mmHg vs 68.4 ± 8.9 mmHg; <0.05), and higher plasma levels of glycaemia (93.6 ± 17.7 mg/dL vs 97.3 ± 26.4 mg/dL; *p* < 0.05) and uric acid (4.8 ± 1.6 mg/dL vs 5.2 ± 2.1 mg/dL; *p* < 0.05). Furthermore, DXM test-positive patients during follow-up showed a significant reduction in the “dipping pattern” phenomenon compared to negative patients (25% vs 50%; *p* < 0.05).

Furthermore, we have compared baseline patients positive at DXM Test with patients positive during follow-up; interestingly, we showed that the latter group showed a statistically lower secretion of cortisol at basal level (407 ± 87 nmol/L vs 519 ± 238 nmol/L *p* < 0.05) and after DXM Test (78 ± 22 nmol/L vs 123.5 ± 23 nmol /L; *p* < 0.05); as well as lower levels of LDL (94.1 ± 21.5 mg/dL vs 115.4 ± 36.5 mg/dL; *p* < 0.05), lower pressure load at the 24-h systolic blood pressure (126.8 ± 12.4 mmHg vs 131.61 mmHg; *p* < 0.05), 24-h diastolic blood pressure (76.6 ± 8.8 mmHg vs 79.7 ± 10 mmHg; *p* < 0.05), diurnal diastolic blood pressure (79.2 ± 8.9 mmHg vs 81.9 ± 11.2 mmHg; *p* < 0.05), and lower dipping pattern (25% vs 41%; *p* < 0.05). To minimise the influence of the pre-laboratory phase, all the blood and urine samples were collected following the same procedure. A peripheral vein was accessed to collect 4 mL of blood in a specific tube containing 18 IU. of lithium heparin per mL of blood. Within one hour, the samples were centrifuged at 2000 rpm for 10 min at 25 °C. Urine samples were collected over a 24-h period (from 7:00 a.m. to 7:00 a.m. the next day) in a sterile container kept in a cool, dark place. Particular care was taken to ensure that no portion of the urine was lost during collection.

## 4. Discussion

Adrenal Incidentaloma (AI) refers to an adrenal mass detected on imaging procedures performed for reasons not related to suspected adrenal disease, particularly apparent in recent decades due to the frequent use of instrumental evaluations. International Guidelines recommend that every patient presenting AI undergo a careful assessment of both the possible malignant nature (primitive of the adrenal gland or as metastatic recurrence) and the possible hormone overproduction [2,10]. Among the primary causes of excessive hormone production —such as primary hyperaldosteronism, pheochromocytoma, hypercortisolism and hyperandrogenism—it is crucial for these patients to undergo a 1-mg overnight DXM suppression test to exclude autonomous cortisol secretion, using plasma cortisol levels post DXM ≤ 50 nmol/L (≤1.8 μg/dL) as a diagnostic criterion for the exclusion of autonomous cortisol secretion [10].

Until 2016, International Guidelines distinguished three categories based on their response to the DXM suppression test: <50 nmol/L (1.8 µg/dL) excluded hypercortisolism; 51–138 nmol/L (1.9–5.0 µg /dL): indicated “possible autonomous cortisol secretion”, and a >138 nmol/L (>5.0 µg/dL) was classified as “autonomous cortisol secretion” [2]. However, recent guidelines consider “mild autonomous cortisol secretion” (MACS) in all patients without signs and symptoms of overt Cushing’s syndrome and post DXM Test plasma cortisol concentration above 50 nmol/L (>1.8 μg/dL), without any further stratification based on the degree of cortisol non-suppressibility. In the literature, several studies confirm the usefulness of the DXM test by the ACTH dosage, while all other additional biochemical tests remain recommended but unnecessary.

**Figure 2:**
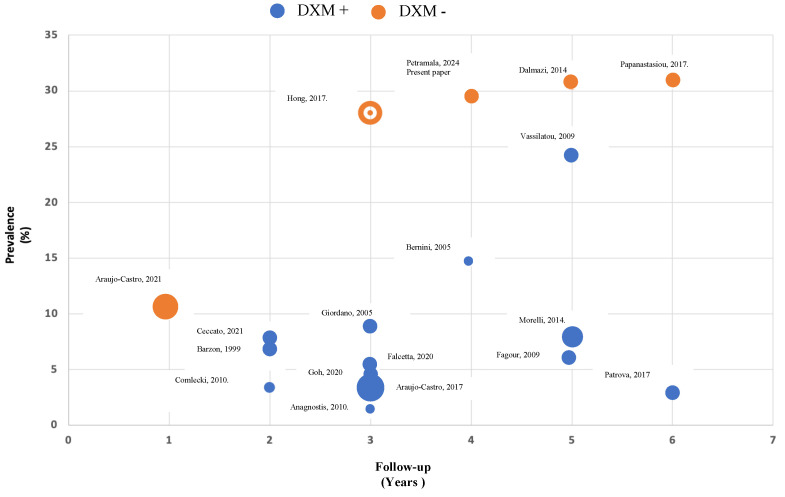
Prevalence of de novo appearance of an autonomous secretion of cortisol in patients with non-functional adrenal incidentaloma (NFAI) during follow-up. Morelli 2014 [11], Dalmazi 2014 [13], Hong 2017 [15], Papanastiou 2017 [16], Comlecki 2010 [18], Ceccato 2021 [19], Falcetta 2020 [20], Bernini 2005 [21], Vassilatou 2009 [22], Araujo-Castro 2021 [23], Giordano 2005 [24], Barzon 1999 [25], Goh 2020 [26], Anagnostis 2010 [27], Fagour 2009 [28], Patrova 2017 [29], Araujo-Castro 2017 [17]. DXM +: MACS diagnosis after positive DXM test plus one abnormal hormonal test of hypothalamic-pituitary-adrenal axis [urinary free cortisol (UFC) level > 100 mcg/24 h; morning plasma ACTH levels <  pg/mL); altered salivary cortisol]; DXM -: MACS diagnosis after positive DXM test alone.

The evaluation of comorbidities potentially attributable to cortisol excess (i.e., arterial hypertension, diabetes mellitus, dyslipidemia, metabolic syndrome, vasculopathy, obesity-related pathologies, and osteoporosis) has a pivotal role in clinical decision-making, in particular concerning the utility of surgical treatment (adrenalectomy) or the advisability of the pharmacological treatment of cortisol-related risk factors [9,12]. Interestingly, the Guidelines recommend against repeated hormonal evaluation in patients with AI and hormonal assessment within the reference range unless new clinical signs of endocrine activity or worsening comorbidities (hypertension, type 2 diabetes, dyslipidemia, onset of a cardiovascular acuity higher). In this regard, in patients with MACS not undergoing adrenalectomy, only annual reassessment of cortisol-reduced comorbidities is recommended [13,14]. For this reason, the usefulness and diagnostic capacity of periodic re-valuation of patients with apparently non-secreting AI (NFAI) is extremely debated in the literature. In the present study, for 5 years follow-up, the possible de novo appearance of an autonomous secretion of cortisol was evaluated in patients with not-functioning AI. In the literature, different authors have evaluated this aspect (Figure 2); the studies carried out using only the DXM test with a cut-off <50 nmol/L (<1.8 μg/dL) have shown a prevalence of MACS detection between 28 and 30.8% [15,16] during observation of 3–6 years, similar to the prevalence found in our study, and a lower prevalence (10.5%) in a study conducted with less follow-up (1 year) [17]. On the other hand, some authors who evaluated the autonomous secretion of cortisol associated through several parameters of the hypothalamic-pituitary-adrenal axis (such as 24-h free cortisol, ACTH, circadian rhythm of salivary cortisol) and lack of suppression on the DXM test showed lower prevalence, between 1% and 24.4%, distinguished in relation to different follow-up (2–6 years) [17,18,19,20,21,22].

In patients with a positive DXM test, both at baseline and during follow-up, compared to NFAI, MACS patients showed higher diastolic blood pressure values, assessed both clinically and through 24-h blood pressure monitoring (24-h, daytime, and nighttime periods). High blood pressure represents one of the main complications of hypercortisolism; therefore, over 60% of patients present a feature of moderate-severe hypertension [30], particularly with a greater load on the diastolic component of blood pressure [31]. Different mechanisms are involved in the development of arterial hypertension and its complications, including increased activity of the renin-angiotensin system through an increase in the hepatic synthesis of angiotensinogen, concomitant up-regulation of angiotensin II type 1A receptors) [32], endotheliopathy is characterised by an imbalance in the vasoregulatory system, reduced bioavailability of vasodilators, increased synthesis of vasoconstrictors such as endothelin 1, and increased vascular reactivity to vasopressors [33]. A further important element is the alteration of the balance between two isoenzymes of 11β-HSD. The 11β-HSD type 1 isoenzyme, a predominant reductase in most intact cells, catalyses the regeneration of active glucocorticoids, metabolising cortisone into its active form cortisol, amplifying cellular action; as regard, 11β-HSD type 1 knockout mice are protected from the effects of GC excess, including hypertension, hepatic steatosis, myopathy and dermal atrophy [34]; while isoenzyme type 2 is responsible for the inactivation of cortisol [35]. In patients with hypercortisolism, tissue expression of 11β-HSD type 1 is overexpressed, while the isoenzyme 11β-HSD type 2 is incapable of inactivating cortisol [36].

Moreover, in MACS patients, at baseline and during follow-up, we have highlighted higher fasting blood glucose and uric acid levels. Hypercortisolism is characteristically associated with the development of obesity and reduced insulin sensitivity, favouring almost 30% of patients with impaired fasting glucose and reduced glucose tolerance until diabetes mellitus [37]. The mechanisms involved are different, direct and indirect; among the latter, it was found that increased free fatty acid production, very-low-density lipoprotein synthesis, hepatic steatosis mediated by overexpression of 11β-HSD type 1, activation of hepatic gluconeogenesis, and adipose tissue structural and functional rearrangement are all at play [30,38,39,40]. Among the mechanisms directly involved between hypercortisolism and hyperglycemia, several studies have described β-cell dysfunction and reduced insulin sensitivity; impaired glucose transporter migration to the cell surface due to post-receptor defect of insulin receptor substrate-1, phosphatidylinositol-3 kinase and protein kinase B in the liver, skeletal muscle and adipose tissue; altered secretion of adipokines, such as adiponectin and leptin, leading to impaired insulin sensitivity [41].

As regarded, previously in adrenal fat of patients with adrenal adenoma cortisol-secreting, we found higher expression of leptin mRNA levels and reduced adiponectin mRNA expression, both strictly related to plasma levels of both of them, suggesting a molecular mechanism favouring dyslipidemia, hyperglycemia, metabolic syndrome and adiposopathy [42].

Some studies have evaluated the relationship between hypercortisolism and plasma uric acid levels. In animal models, GR activation induces increased expression of the enzyme responsible for increased production of uric acid (Xanthine Oxidase) at the muscle level [43] and altered kidney elimination through downregulation of mRNA levels of URAT1 [44]. Finally, since hypercortisol induces an increased expression of visceral fat, this could interfere with increased plasma levels of uric acid.

Interestingly, we found that patients with a positive DXM test during follow-up showed lower values of blood pressure (24-h systolic and diastolic and daytime diastolic assessed by ABPM) and LDL cholesterol levels compared to patients with a positive DXM test at baseline. Screening with the DXM test is carried out at the AI diagnosis, but we do not know precisely the onset of autonomous cortisol secretion. So, these different clinical manifestations may be related to the different times of exposure to hypercortisolism.

As limitations of the study, we did not dose plasma levels of dexamethasone; this aspect would be useful to verify the true positives/negatives; it is not a mandatory aspect according to the guidelines; moreover, we did not measure cortisone levels, a useful element for verifying the activity of the HSD11 isoenzymes (B1 and B2); these limitations could be addressed in future studies.

An innovative element of our study was to underline that the follow-up of AI patients allows the early identification of those patients in whom an autonomous secretion of cortisol occurs, allowing a pharmacological therapy aimed at reducing the factors of cardiovascular and metabolic risk and greater clinical control.

## Figures and Tables

**Table 1:** Demographic and hemodynamic data of the study groups.

Baseline	Age (Years)	Sex (M/F)	BMI (kg/m^2^)	WC (cm)	SBP (mmHg)	DBP (mmHg)	HR (bpm)
**All** **patients (N = 132)**	61.7 ± 10.8	28/38	27.6 ± 3.9	98.8 ± 12.0	135.5 ± 15.3	82.2 ± 10.1	67.4 ± 9.7
**Negative DXM T0 (N = 89)**	61.6 ± 11.5	17/27	27.6 ± 4.4	98.7 ±1 3.0	135.8 ± 16.7	80.6 ± 9.9	68.4 ± 9.2
**Positive DXM T0 (N = 43)**	61.8 ± 9.4	11/11	27.7 ± 2.7	99.1 ± 10.3	134.9 ± 13.2	85.6 ± 8.47	65.5 ± 10.3
***p*-value**	ns	ns	ns	ns	ns	<0.05	ns
**Follow-up**	**Age** **(years)**	**Sex** **(M/F)**	**BMI** **(kg/m^2^)**	**WC** **(cm)**	**SBP** **(mmHg)**	**DBP** **(mmHg)**	**HR** **(bpm)**
**Negative DXM T1 (N = 63)**	64.6 ± 11.7	10/18	28.0 ± 4.7	100.9 ± 16.3	134.3 ± 14.8	81.6 ± 10.5 *	65.6 ± 8.5
**Positive DXM T1 (N = 26)**	62.2 ± 8.62	7/9	27.9 ± 4.2	101.1 ± 11.7	134.3 ± 22.8	83.7 ± 9.7 *	66.9 ± 11.7
***p*-value**	ns	ns	ns	ns	ns	<0.05	ns
***p*-value** **Positive T0 DXM vs Positive T1 DXM**	ns	ns	ns	ns	ns	ns	ns

BMI: body mass index; WC: waist circumference; SBP: systolic blood pressure; DBP: diastolic blood pressure; HR: heart rate; T0: baseline; T1: end of follow-up, * statistical significant difference.

**Table 2:** Ambulatory blood pressure monitoring (ABPM) data of the study groups.

Baseline	SBP-24 h (mmHg)	DBP-24 h (mmHg)	HR-24 h (bpm)	SBP-D (mmHg)	DBP-D (mmHg)	HR-D (bpm)	SBP-N (mmHg)	DBP-N (mmHg)	HR-N (bpm)	Dipper %
**All** **patients (N = 132)**	130.3 ± 13.6	77.1 ± 10.0	73.5 ± 7.7	133.0 ± 14.3	80.0 ± 10.5	75.6 ± 7.7	121.3 ± 14.1	69.2 ± 9.3	66.4 ± 7.6	54
**Negative DXM T0 (N = 89)**	129.7 ± 15.3	75.9 ± 9.6	73.9 ± 7.9	132.1 ± 16.3	78.9 ± 10.3	76.5 ± 7.4	121.3±14.9	67.8 ± 8.5	66.6 ± 7.4	60
**Positive DXM T0 (N = 43)**	131.6 ± 10.8	79.7 ± 10 *	72.9 ± 6.8	134.7 ± 0.6	81.9 ± 11.2 *	72.4 ± 7.2	121.4 ± 13.0	72.4 ± 10.8 *	66.1 ± 7.9	41
***p*-value**	ns	<0.05	ns	ns	<0.05	ns	ns	<0.05	ns	ns
**Follow-up**	**SBP-24 h** **(mmHg)**	**DBP-24** **h** **(mmHg)**	**HR-24 h** **(bpm)**	**SBP-D** **(mmHg)**	**DBP-D** **(mmHg)**	**HR-D** **(bpm)**	**SBP-N** **(mmHg)**	**DBP-N** **(mmHg)**	**HR-N** **(bpm)**	**Dipper** **%**
**Negative DXM T1 (N = 63)**	125.1 ± 11.7	71.3 ± 6.9	73.2 ± 7.4	127.5 ± 10.9	73.9 ± 7.3	74.5 ± 7.6	113.3 ± 26.5	64.5 ± 7.2	67.4 ± 8.6	50
**Positive DXM T1 (N = 26)**	126.8 ± 12.4	76.6 ± 8.8 *	74.7 ± 8.9	129.6 ± 14.8	79.2 ± 8.9 *	75.5 ± 9.2	117.8 ± 15.2	68.4 ± 8.9 *	69.7 ± 11.1	25
***p*-value**	ns	<0.05	ns	ns	<0.05	ns	ns	<0.05	ns	<0.05
***p*-value** **Positive DXM T0 vs Positive DXM T1**	<0.05	<0.05	ns	ns	<0.05	ns	ns	ns	ns	<0.05

SBP-24 h, 24-h systolic blood pressure; DBP-24 h, 24-h diastolic blood pressure; HR-24 h, 24-h heart rate; SBP-D, diurnal systolic blood pressure; DBP-D, diurnal diastolic blood pressure; HR-D, diurnal heart rate; SBP-N, nocturnal systolic blood pressure; DBP-N, nocturnal diastolic blood pressure; HR-N, nocturnal heart rate; T0: baseline; T1: end of follow-up, * statistical significant difference.

**Table 3:** Hormonal data of the study groups.

Baseline.	Plasma Cortisol (nmol/L)	Test DXM (nmol/L)	Plasma ACTH (pg/mL)	Urinary Free Cortisol (nmol/24 h)	Maximum Diameter Adrenal Adenoma (mm)
**All patients (N = 132)**	434 ± 252	54.6 ± 85	11.1 ± 5.4	148 ± 102	
**Negative DXM T0 (N = 89)**	354 ± 224	20.1 ± 18	9.2 ± 2.3	134 ± 83	27.17 ± 7.41
**Positive DXM T0 (N = 43)**	519 ± 238	123.5 ± 23	12.2 ± 3.9	174 ± 50	27.48 ± 7.59
***p*-value**	<0.05	<0.001	ns	<0.02	ns
**Follow-up**	**Plasma cortisol** **(nmol/L)**	**Test DXM** **(nmol/L)**	**Plasma ACTH** **(pg/mL)**	**Urinary free cortisol** **(nmol/24 h)**	**Maximum diameter adrenal adenoma (mm)**
**Negative DXM T1 (N = 63)**	331 ± 172	24.2 ± 21	9.6 ± 3.4	125 ± 65	28.31 ± 8.81
**Positive DXM T1 (N = 26)**	407 ± 87	78 ± 22	9.9 ± 2.8	145 ± 76	28.74 ± 9.21
***p*-value**	<0.05	<0.001	ns	ns	ns
***p*-value** **Positive DXM T0 vs Positive DXM T1**	<0.05	<0.05	ns	ns	ns

DXM Test: 1 mg Dexamethasone; T0: baseline; T1: end of follow-up.

**Table 4:** Biochemical parameters, blood glucose and lipid profile data of the study groups.

Baseline	Creatinine (mg/dL)	Glycaemia (mg/dL)	LDL (mg/dL)	Triglycerides (mg/dL)	Uric acid (mg/dL)	μ-Albuminuria (24 h)
**All** **patients (N = 132)**	0.89 ± 0.24	96.6 ± 19.9	113.5 ± 37.5	124.7 ± 52.9	5.0 ± 1.6	24.2 ± 15.2
**Negative DXM T0 (N = 89)**	0.85 ± 0.20	94.6 ± 19.8	114.1 ± 37.6	133.6 ± 55.8	4.7 ± 1.6	23.8 ± 15.0
**Positive DXM T0 (N = 43)**	0.99 ± 0.32	97.4 ± 18.7	115.4 ± 36.5	112.3 ± 41.4	5.4 ± 1.7	23.1 ± 10.8
***p*-value**	ns	<0.05	ns	ns	<0.05	ns
**Follow-up**	**Creatinine** **(mg/dL)**	**Glycaemia** **(mg/dL)**	**LDL** **(mg/dL)**	**Triglycerides** **(mg/dL)**	**Uric acid** **(mg/dL)**	**μ-Albuminuria** **(24 h)**
**Negative DXM T1 (N = 63)**	0.92 ± 0.22	93.6 ± 17.7	98.6 ± 24.5	127.9 ± 59.8	4.8 ± 1.6	21.3 ± 48.7
**Positive DXM T1 (N = 26)**	0.84 ± 0.29	97.3 ± 26.4	94.1 ± 21.5	138.6 ± 100.1	5.2 ± 2.1	27.8 ± 55.6
***p*-value**	ns	<0.05	ns	ns	<0.05	ns
***p*-value** **Positive DXM T0 vs Positive DXM T1**	ns	ns	<0.03	ns	ns	ns

LDL: LDL-cholesterol; T0: baseline; T1: end of follow-up.

## Data Availability

The raw data supporting the conclusions of this article will be made available by the authors without undue reservation.
